# GOING FOR THE WIN-WIN-WIN: HARNESSING THE POWER OF SENIOR VOLUNTEERISM TO ADDRESS DEMENTIA CARE AND PROMOTE HEALTH

**DOI:** 10.1093/geroni/igz038.2925

**Published:** 2019-11-08

**Authors:** Quincy M Samus, Joseph E Gaugler, George W Rebok

**Affiliations:** 1 The Johns Hopkins University, Baltimore, Maryland, United States; 2 University of Minnesota - School of Public Health, Division of Health Policy and Management, Minneapolis, Minnesota, United States; 3 Johns Hopkins Bloomberg School of Public Health, Baltimore, Maryland, United States

## Abstract

The public health implications of Alzheimer’s disease or related dementias (ADRDs) are significant and have placed considerable pressure on the U.S. healthcare system. Training and mobilizing a critical mass of volunteers to address unmet dementia care needs may be a potent, scalable, and cost efficient approach to address gaps in dementia care and to support family caregivers. Further, by engaging older volunteers to do this work and remain in productive and impactful post-retirement roles, additive population health benefits may be possible. This session will focus on ways we might harness the power of senior volunteers to meet the public health challenges associated with ADRD. Presentations will draw from three innovative community-based projects that utilize senior volunteers to support and enhance health in aging and dementia care. Dr. Carlson will provide an update on the evaluation and scaling Experience Corps, an intergenerational program that engages senior volunteers to work in elementary schools. Dr. Gaugler will discuss the Porchlight Project, a new multicomponent training approach for senior volunteers in Minnesota to enhance dementia care capabilities and support to underserved older persons. Dr. Samus will introduce the MEMORI Corps program, a novel activity-based companion care program for home-residing persons with ADRD delivered by trained senior volunteers. Given the current and impending shortages in the geriatric work force and family caregivers, respectively, innovative and readily available long term service and support options are needed to offset potential care gaps. The current session proposes the novel incorporation of volunteers as one solution to do so.

